# Cognition and driving in older adults: a complex relationship

**DOI:** 10.18632/aging.204551

**Published:** 2023-02-21

**Authors:** Luis Eudave, María A. Pastor

**Affiliations:** 1 Faculty of Education and Psychology, University of Navarra, Spain

**Keywords:** driving simulator, visual cognition, distance perception, fMRI

Driving is a complex daily activity that requires several cognitive abilities, such as cognitive fitness, perception, attention, executive functions, and memory, in order to be successful. As we age, some of these abilities may deteriorate, such as attention or executive functions, however, research has found that only deficits in visuospatial abilities are consistently associated with impaired driving in older adults [1, 2]. Understanding how changes in cognition affect driving performance in older populations can lead to the development of cognitive screening protocols that can quickly detect deficits and prevent fatal or injury-inflicting accidents. Additionally, it is not yet clear what brain mechanisms involved in driving-related cognition affect actual driving performance and outcomes.

Recently [3], we examined the age-related differences in how egocentric distance perception (EDP) impacts driving in a simulator and its neural correlates, as measured by functional magnetic resonance imaging (fMRI). We chose to further explore how EDP changes with aging since previous studies have shown mixed results. To do this, we adapted the task to a more naturalistic driving scenario that could be examined using fMRI. Therefore, the EDP task used in this experiment involved calculating distances between a car from the driver’s point of view (left cockpit) and another vehicle located in front of it. Results showed that, despite higher response times, older adults were able to accurately perceive distances when compared to a younger driver group, consistent with a small majority of studies on EDP.

Imaging results revealed that in older adults, there is an increase in activity in prefrontal and parietal regions, along with higher functional connectivity between frontal and parietal, occipital, and cerebellar nodes. No studies had previously explored this effect in older populations, but these results align with those describing how other cognitive abilities are maintained with age at the “cost” of additional and/or alternate patterns of brain activation or changes in functional connectivity. This mechanism could be explained by either a dedifferentiation of cortical systems in charge of perceptual information that become less distinctive as we grow older (for a review see [4]) or due to a compensatory response that aims to sustain performance by recruiting additional neural resources (for a recent perspective see [5]).

Our driving simulator collected vehicle telemetry data (steering, braking, speeding, etc.,) as well as other driving outcomes (lane keeping, response to traffic lights, etc.,) from a dynamic scenario that included driving on the highway, a mountain road, and the city. Consistent with previous findings (for a review see [6]) in real-world and simulated driving tasks, older adults drove at slower speeds than their younger counterparts, independently of the speed limits in each segment. They also braked less, skipped more yellow traffic lights, and invaded sidewalks more frequently (significance was lost after multiple comparisons adjustments); the latter might be related to the previously observed decrease in lane keeping. In general, we argue that by reducing driving speed, older drivers are able to prevent challenging situations and commit fewer mistakes, at least when they are not distracted.

Finally, we explored how performance in the EDP task was related to driving behaviors. Interestingly, our analysis showed that accuracy was negatively correlated with dangerous braking behaviors in a driving simulator (those with higher accuracy had fewer sudden hard braking behaviors). Faster braking times have been associated with better general cognitive performance [7]; however, this effect might be hindered in older adults by the presence of visual distractors [8].

This study hopes to add evidence to a field where the number of studies evaluating cognition and its neural correlates driving in older drivers remains scarce. Several factors may help explain this gap: most driving studies, in general, include mostly young and/or middle-aged adults; the inherent difficulties of real-world and simulated driving tasks and neuroimaging studies become more evident with older adults (for example, cybersickness or claustrophobia); the added difficulty in finding the appropriate candidates that fill in all inclusion criteria and that are willing to participate in a several-session study. All of these contribute to the low number of studies and their small sample sizes, an instance that brings its own set of limitations.

As the world’s population continues to age, it becomes ever more important to characterize how cognition is involved in driving. It is clear that normal cognitive aging impairs some older adults’ driving ability. However, we lack complete knowledge of the underlying processes behind those impairments and, perhaps more importantly, the resources to systematically assess fitness to drive. We encourage researchers to incorporate neuroimaging techniques (fMRI, EEG, fNIRS) for the evaluation of cognition in older drivers, as well as to collaborate and share materials and data, whenever possible, to improve the generalizability of results. If followed, these efforts can lead to the development of better tools for predicting driving performance, detecting unsafe driving behaviors and, ultimately, preventing motor vehicle accidents.
